# Chronic Kidney Disease Education Class Improves Rates of Early Access Creation and Peritoneal Dialysis Enrollment

**DOI:** 10.7759/cureus.21306

**Published:** 2022-01-16

**Authors:** Saud A Aloudah, Bandar A Alanazi, Mohammed A Alrehaily, Abdulrahman N Alqessayer, Nawaf S Alanazi, Elwaleed Elhassan

**Affiliations:** 1 College of Medicine, King Saud bin Abdulaziz University for Health Sciences, Riyadh, SAU; 2 Department of Nephrology, King Abdulaziz Medical City, Riyadh, SAU

**Keywords:** educational class, renal transplantation, general nephrology dialysis and transplanation, peritoneal dialysis (pd), hemodialysis, end stage renal disease (esrd)

## Abstract

Background

Most patients with end-stage kidney disease begin hemodialysis (HD) in an unplanned fashion at a late stage, necessitating the commencement of HD with a temporary venous catheter, the least favorable option. Alternative modalities of kidney replacement therapy (KRT), peritoneal dialysis (PD), and preemptive transplant offer similar or better outcomes than HD at a lower overall cost, and yet they remain underutilized in Saudi Arabia. Early education may help prepare patients with advanced chronic kidney disease (CKD IV and V) to accept their disease and choose a KRT modality that minimizes complications and matches their lifestyle.

The aim of the study is to assess the impact of a pilot educational class on therapy choices and outcomes.

Methodology

In a cross-sectional study, we conducted phone interviews and reviewed medical records of 81 attendees of the multidisciplinary monthly educational class about KRT that was held at the King Abdulaziz Medical City (KAMC) from January 2017 to October 2021. The interview was conducted at least one year after the participants attended the class. The study proposal, consent, and questionnaire were approved by the King Abdulaziz International Medical Research Center. Patient data was retrieved from KAMC electronic medical record system.

Results

Volunteer participation in the survey was high (62/81). For the respondents, a preemptive kidney transplant was the most preferred (48/62, 77%) option for KRT. Among the preferred fallback options, HD was the most frequently chosen (29/62, 47%) compared to PD (26/62, 41.9%). At the time of the interview, a great majority of the patients (54/62, 87%) was already on KRT, including about half (26/54, 48%) on HD via a catheter, and the rest about equally divided between those on HD via an arteriovenous (AF) fistula (13/54, 24%) and those on PD (15/54, 28%). Thus, half of the respondents on KRT (28/54, 51%) avoided urgent HD catheter commencement. However, because of an unfortunate shortage of donors, only a small minority (2/62, 3%) of patients received preemptive transplantation.

Conclusion

The KAMC CKD education class helped boost the fraction of patients, significantly above the national average, who accepted the diagnosis of kidney failure and pursued preemptive native HD access or enrolled in PD.

## Introduction

Most patients with end-stage kidney disease (ESKD) begin dialysis in an unplanned fashion dictating initiation via a temporary catheter [1], which is rife with complications [2]. Although alternative modalities of kidney replacement therapy (KRT) may support similar or better patient outcomes with other benefits, these are, unfortunately, underutilized in Saudi Arabia [3]. Most Saudi nephrologists believe that peritoneal dialysis (PD) should be offered to chronic kidney disease (CKD) patients as the first dialysis modality [4]. The efficacy of hemodialysis (HD) and PD is the same, but PD is considered more cost-effective [5]. In addition, the quality of life for a CKD patient is better when undergoing PD than HD [6]. A retrospective cohort study showed that PD is considered a better choice of KRT in young patients [7]. A survey of Saudi ESKD patients undergoing HD suggested that their refusal of PD is related to a lack of prior and adequate counseling and education about PD [8]. Moreover, the preferred type of vascular access for chronic HD is arteriovenous (AV) fistula rather than HD catheters [9]. HD catheter use has been consistently associated with worse rates of complication and survival [8]. According to the Dialysis Outcomes and Practice Patterns Study (DOPPS), an international prospective cohort study, the use for HD initiation of arteriovenous access is lowest (at 19%), and that of central venous catheter is highest (at 81%) in Gulf Cooperation Council (GCC) member countries [10]. Taken together, these studies suggest that advanced CKD patients in these countries are not given adequate time and resources to make informed decisions about their optimal KRT options. Early education may help CKD patients acknowledge their disease and choose a KRT modality that minimizes complications and matches their lifestyle [11]. In fact, early education has been shown to increase the proportion of patients initiating dialysis with PD [12]. These precedents motivated us to start a pilot multidisciplinary educational class at KAMC for patients with advanced CKD. We have previously reported on the attendees’ high level of satisfaction with the class [13].

## Materials and methods

Patient education

Nephrology staff extended invitations to all patients with advanced CKD attending KAMC outpatient nephrology clinics and encouraged them to attend the class. KAMC is a large tertiary care center in Riyadh serving members of the National Guard and their families across Saudi Arabia. The two-hour monthly class was delivered by a team comprising a nephrologist, dialysis nurses, a dietician, and vascular and transplant coordinators in a roundtable fashion. For background and context, it featured an overview of normal kidney function, pathophysiological alterations, and medical and nutritional management of CKD. Then patients and their family members learned about the benefits, complications, and outcomes of different KRT options, which were presented in a balanced manner and with emphasis on early choice and access planning. Brochures, sample catheters, and a PD mannequin were made available for demonstration. Participants were given ample opportunity to ask questions and received the contact information of the PD Unit and the coordinators for assistance with their further management. After attending the educational class, patients were sent back to their primary nephrologists to map their next move in the process.

Cross-sectional data

The study took place at the KAMC PD unit. After one year of attending the class, each patient was queried about their choices and outcomes using a questionnaire. Every class attendee was included and individually contacted by phone. The information they provided was corroborated by cross-checking with the hospital medical record system (BestCare; ezCaretech, Seoul, South Korea). Variables retrieved from the hospital database included age, gender, height, weight, body mass index (BMI), and baseline glomerular filtration rate (GFR). Variables obtained from the patient in the questionnaire included marital status, occupation, educational level, and the patients’ preferred KRT modality upon finishing the class. In addition, patients were asked about their outcomes at the time of the questionnaire (i.e., one year or more time past their class) and whether they had been commenced on any form of KRT. For those who started dialysis, commencing type of access and reasons for not undergoing transplantation were also recorded. The consent form and questionnaire can be found in the appendix.

Data Analysis

Data entry and statistical analysis were done by SPSS version 21 (IBM Inc., Armonk, New York). Frequencies and percentages were calculated for categorical data, such as “preferred choice of treatment.”

## Results

Patient Demographics

Of 81 class attendees invited in the study, 62 returned complete responses (15 declined; four were deceased). Demographics of the patient pool have been summarized in Table 1. Participants’ ages ranged broadly (15 to 85 years), with the majority in the middle-age (46 through 65) cohort. The majority were male (36/62, 58%), married (76%), not active (69.3% unemployed or retired), had some education (69%), and, notably, were overweight or obese (71%).

**Table 1:** Demographics of 62 CKD patients CKD - chronic kidney disease

		Number	Percentage
Gender	Male	36	58.1%
	Female	26	41.9%
Age groups	15 to 35	14	22.6%
	36 to 45	8	12.9%
	46 to 65	22	35.5%
	66 to 85	18	29.0%
Marital status	Single	11	17.7%
	Married	47	75.8%
	Divorced	1	1.6%
	Widowed	3	4.8%
Occupation status	Employed	13	20.9%
	No job	24	38.7%
	Retired	19	30.6%
	Student	6	9.7%
Level of education	Illiterate	19	30.6%
	Basic education	23	37.1%
	High education	20	32.3%
BMI category	Underweight	4	6.5%
	Normal	14	22.6%
	Overweight	25	40.3%
	Obese	19	30.6%

Preferred KRT

Patients were asked to rank their order of KRT preference (Table 2). At the time of the interview, at least one year had passed since the patient attended the class. The preferred first choice was preemptive kidney transplant for the majority (48/62, 77%), followed by HD (9/62, 15%). Apparently, PD was the least popular first choice (5/62, 8%).

**Table 2:** Post-class preference for kidney replacement therapy

		Number	Percentage
First preferred choice of treatment	Hemodialysis	9	14.5%
	Peritoneal dialysis	5	8.1%
	Preemptive transplant	48	77.4%
Second preferred choice of treatment	Hemodialysis	29	46.8%
	Peritoneal dialysis	26	41.9%
	Preemptive transplant	7	11.3%

Patients were also asked to pick an alternative modality if their first choice was not feasible. Among the fallback options, hemodialysis was most popular (29/62, 47%), followed by PD (26/62, 42%) and preemptive transplantation (7/62, 11%). This order of preference is a shifted version of the primary preferences, providing indirect proof of the patients’ consistency and deliberation in choosing their preferred options.

Actual KRT

Information derived from the patient questionnaire and the hospital record system was used to determine patient outcomes after an interval of one year or longer passed since they attended the class. The data is summarized in Table 3. A large majority (54/62, 87%) of patients were already put on KRT, including 39/54 (72%) on HD and 15/54 (28%) on PD. Unfortunately, two-thirds of those in hemodialysis therapy were commenced by a temporary catheter (26/39, 67%) rather than a native AV fistula (13/39, 33%). On the positive side, all 15 patients on PD therapy had skipped interim HD. Thus, half of the patients on KRT, 28/54 (51%), avoided urgent HD catheterization, the commencement mode most rife with complications.

**Table 3:** Patient outcomes one plus years after class AV - arteriovenous

Outcome	Number	Percentage
Still under medical observation	6	9.7%
Hemodialysis via a temporary catheter	26	41.9%
Hemodialysis via an AV fistula	13	21.0%
Peritoneal dialysis	15	24.2%
Preemptive kidney transplant	2	3.2%

Despite preemptive transplant being the most preferred choice of KRT in our patient group, only a small minority (2/62, 3%) of the respondents were lucky enough to have received a transplant kidney by the time of the interview, while another minority (6/62, 10%) were still undergoing medical observation. Unavailability of donors was the highest reported (15/40, 38%) reason for not attaining a transplant.

## Discussion

Denial and misinformation commonly afflict CKD patients’ thinking about their disease. Moreover, many present for KRT in an advanced stage, necessitating an urgent and unplanned commencement on hemodialysis via a temporary intravenous catheter. Preemptive transplantation and PD can offer similar or better outcomes, yet these KRT options are distinctly underutilized in Saudi Arabia. Notably, the fraction of CKD patients on some form of KRT who received PD in the Kingdom was minuscule (1416/23,728, 6%) in 2014 [2].

Patient education has been shown to help significantly improve KRT outcomes. A structured, patient-centered education program significantly increased the frequency of opting for PD in patients needing unplanned KRT in Germany [14]. A retrospective analysis conducted in South Korea, using propensity score matching, showed that patients who received multidisciplinary pre-dialysis education, compared with those who did not, commenced dialysis at a higher GFR and had less need for urgent unplanned dialysis [15]. Those precedents motivated us to run a multidisciplinary education program for patients with late-stage CKD in the Kingdom to educate them about their disease, its natural progression, and the full spectrum of KRT options. The program put special emphasis on early access and the benefits of PD and preemptive kidney transplantation over HD.

In this survey, we compare the patients’ initial preferences with their documented outcomes one year after attending the class or later. Unsurprisingly, most patients chose preemptive transplant as their first preference. However, medical ineligibility or shortage of donors usually prevent the realization of that preference for the vast majority. In our survey, only three out of 48 (6%) who had preemptive transplant as their first preference did actually receive it.

Remarkably, more than 40% of the class attendees indicated PD as their second (fallback) option. This corroborates surveys conducted elsewhere that reported PD could be popular if it is presented to pre-dialysis patients in a balanced and thorough manner. A quarter of the patients in our survey ended up receiving PD, and 100% of the latter skipped an interim urgent HD phase. This is a dramatic improvement over the current national average PD utilization (<10% of all KRT) in the Kingdom, underscoring the value of patient education in raising awareness of PD and facilitating patient recruitment for PD [4].

Hemodialysis was the most utilized method for KRT initiation. Unfortunately, two-thirds of those who began HD received it through a temporary catheter, the least desirable option. Native arteriovenous fistula (AVF), which is safer and more secure, was created in only one-third of HD patients in our sample. Although this is still far from optimal, it is higher than those reported for GCC countries in the DOPPS study survey [10].

Our survey was designed as an interventional study aimed to improve the clinical outcome in our own clinical routine through pre-dialysis patient education. The results, although preliminary obtained in a small sample, are highly encouraging and are consistent with precedents reported in other countries. However, because of its observational design, our study is highly specific to the local Saudi patient population, and its applicability in broader demographics has limits. In addition, group allocation was not performed in a randomized con­trolled setting, thus confounding effects cannot be excluded. Lastly, the measures we report for successful outcomes may have a possible bias from volunteer participation (patients who declined may represent a group with less optimal outcomes), which can only be excluded using a longer-term retrospective survey on a much larger patient pool.

## Conclusions

In conclusion, survey measures indicate that our patient education class succeeded as attendees had a significantly higher proportion of accepting the diagnosis of kidney failure, and a significantly higher fraction pursued preemptive native HD access or enrolled in PD than the national average.

## Figures and Tables

**Figure 1:**
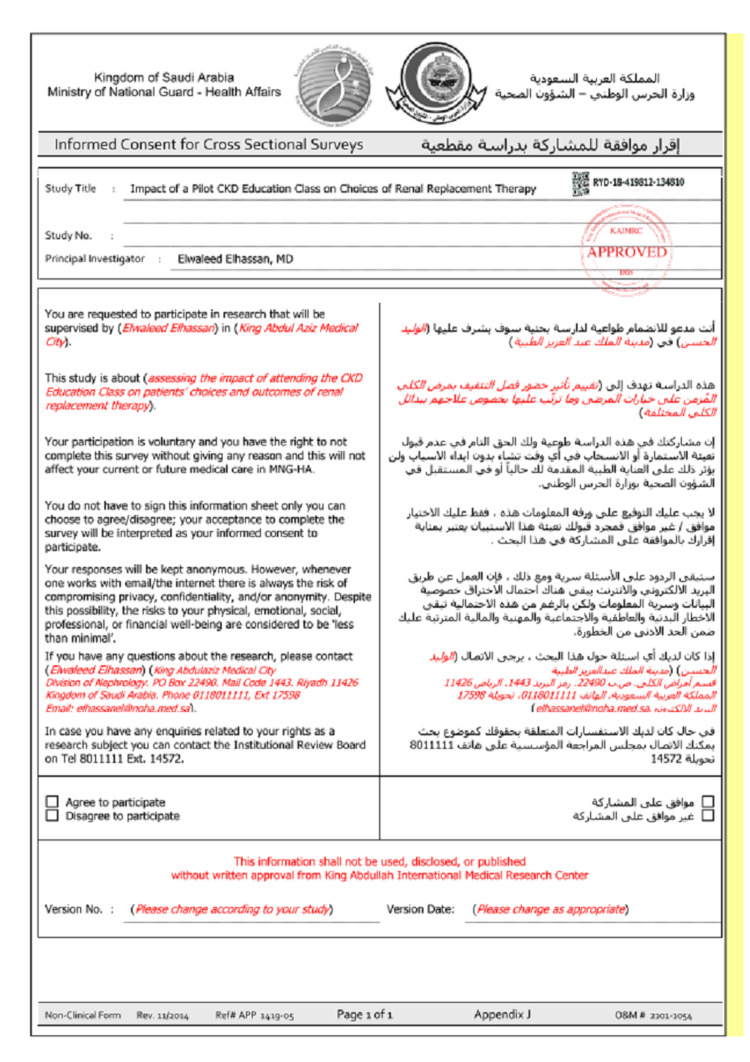
Consent form

**Figure 2:**
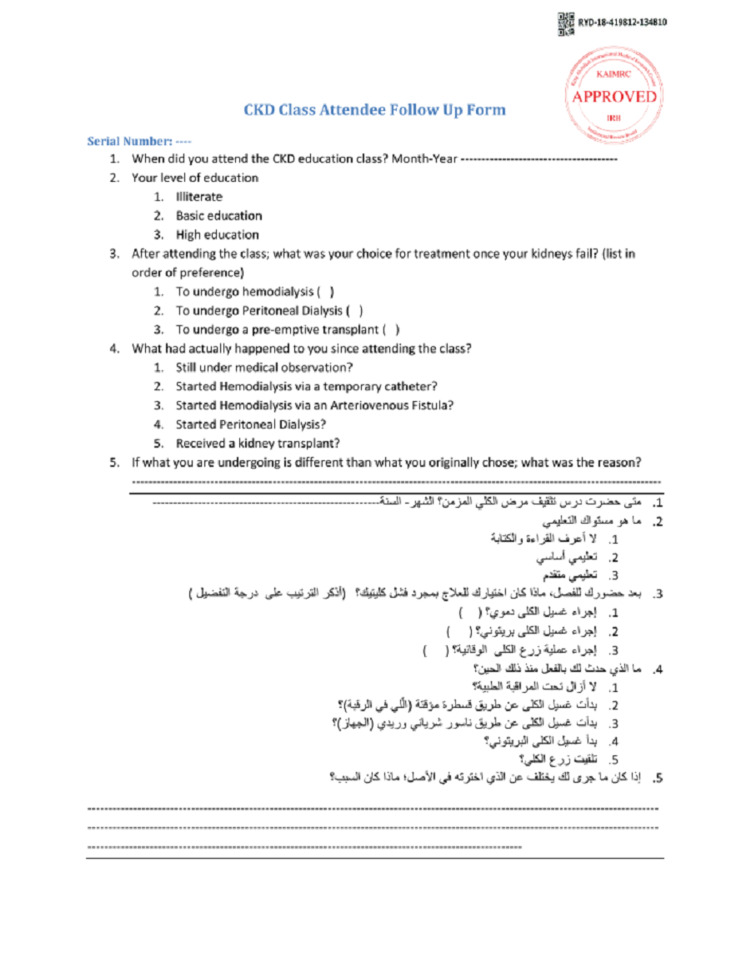
Questionnaire
